# The Inert Brain: Explaining Neural Inertia as Post-anaesthetic Sleep Inertia

**DOI:** 10.3389/fnins.2021.643871

**Published:** 2021-03-02

**Authors:** Andrea I. Luppi, Lennart R. B. Spindler, David K. Menon, Emmanuel A. Stamatakis

**Affiliations:** ^1^Division of Anaesthesia, School of Clinical Medicine, University of Cambridge, Cambridge, United Kingdom; ^2^Department of Clinical Neurosciences, University of Cambridge, Cambridge, United Kingdom; ^3^Wolfson Brain Imaging Centre, University of Cambridge, Cambridge, United Kingdom

**Keywords:** neural inertia, sleep inertia, anaesthesia, orexin, noradrenaline, anticorrelations, aging

## Abstract

“Neural inertia” is the brain’s tendency to resist changes in its arousal state: it is manifested as emergence from anaesthesia occurring at lower drug doses than those required for anaesthetic induction, a phenomenon observed across very different species, from invertebrates to mammals. However, the brain is also subject to another form of inertia, familiar to most people: sleep inertia, the feeling of grogginess, confusion and impaired performance that typically follows awakening. Here, we propose a novel account of neural inertia, as the result of sleep inertia taking place after the artificial sleep induced by anaesthetics. We argue that the orexinergic and noradrenergic systems may be key mechanisms for the control of these transition states, with the orexinergic system exerting a stabilising effect through the noradrenergic system. This effect may be reflected at the macroscale in terms of altered functional anticorrelations between default mode and executive control networks of the human brain. The hypothesised link between neural inertia and sleep inertia could explain why different anaesthetic drugs induce different levels of neural inertia, and why elderly individuals and narcoleptic patients are more susceptible to neural inertia. This novel hypothesis also enables us to generate several empirically testable predictions at both the behavioural and neural levels, with potential implications for clinical practice.

## Introduction

### Anaesthesia and Sleep

General anaesthesia refers to a pharmacological intervention designed to produce a state of controlled and reversible unconsciousness and unresponsiveness to sensory stimulation. Its discovery is among the greatest in medical history: it allows surgeons to perform millions of life-saving interventions every year, which would be otherwise impossible or extremely distressing.

However, the mechanisms of anaesthetic action in the brain remain incompletely understood—especially since multiple anaesthetic drugs exist, with different pharmacological profiles (Scharf and Kelz, 2013). Nevertheless, anaesthesia is not the only way in which one can become unconscious: the brain exhibits a strong need for periodic unconsciousness in the form of sleep, with the average human spending about a third of their life in this state. A sleep-like state of rapidly reversible physical quiescence, with elevated thresholds to sensory stimulation, has been identified in most species, including even insects (Shaw, 2000) and nematodes (Raizen et al., 2008).

**FIGURE 1:**
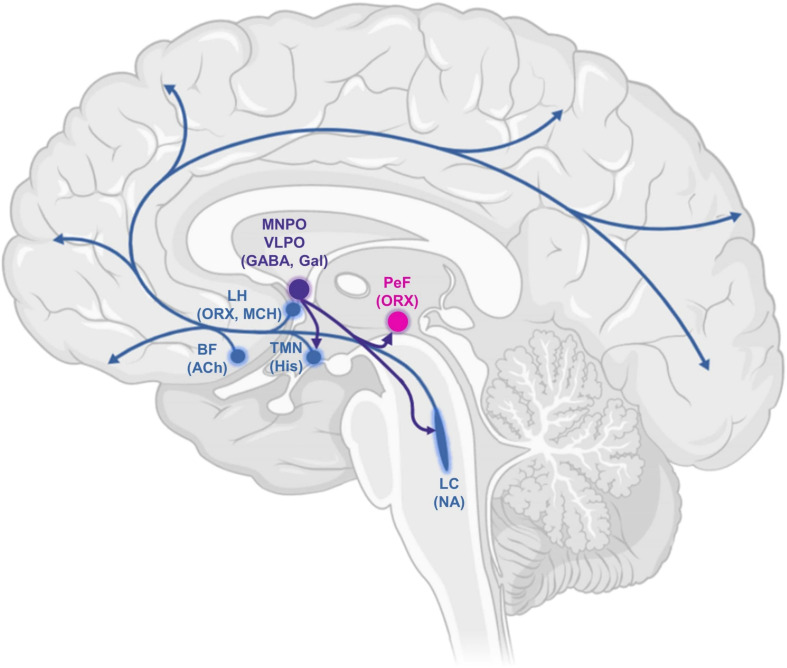
Schematic drawing of some key components of the ascending arousal system, highlighting projections of the ventrolateral preoptic area. This comprises cortical projection neurons originating from the basal forebrain (BF); the recently characterised orexin/hypocretin neurons in the lateral hypothalamus (LH); perifornical orexin neurons (PeF); and several monoaminergic nuclei: the noradrenergic locus coeruleus (LC), the histaminergic tuberomammillary nucleus (TMN) and the ventrolateral preoptic area (VLPO) and median preoptic area (MNPO). Serotonergic and dopaminergic components are not shown. MCH, melanin-concentrating hormone; Gal, galanin; Ach, acetylcholine; ORX, orexin; His, histamine; NA, noradrenaline.

In addition to behavioural similarities with sleep, several anaesthetic drugs generate EEG rhythms that resemble those observed during different stages of sleep: halothane and isoflurane produce a theta rhythm (5–9 Hz) reminiscent of rapid eye movement (REM) sleep (Pang et al., 2009), whereas the GABA-ergic agent propofol and the α2-adrenoreceptor agonist dexmedetomidine induce slow-wave activity (<4 Hz) analogous to what is observed during non-REM (NREM) sleep (Gent and Adamantidis, 2017). Given the behavioural and electrophysiological similarities between sleep and the effects of several anaesthetic agents, the neuronal circuitry underlying sleep may provide critical insights into the mechanisms of anaesthetic action (Karan et al., 2007), with evidence that at least some anaesthetics do in fact intervene on sleep-wake regulating neurons, especially in hypothalamic areas (Franks, 2008; Zecharia et al., 2009; Zhang et al., 2015; Gent and Adamantidis, 2017)—although it should be noted that this similarity is not universal: some other anaesthetics produce desynchronised EEG with little resemblance to sleep EEG, e.g., ketamine, benzodiazepines (Gent and Adamantidis, 2017). The function of sleep is only partly understood, and several different theories have been put forward to explain the existence of this peculiar state (Vyazovskiy, 2015; Joiner, 2016; Krueger et al., 2016), including energy restoration (Berger and Phillips, 1995; Schmidt, 2014) memory consolidation (Abel et al., 2013) and synaptic homeostasis (Tononi and Cirelli, 2014, 2016). Nevertheless, the brain circuits that control sleep are relatively well understood: a wake-promoting and a sleep-promoting system interact in the brain (Figure 1; Saper et al., 2005; Luppi, 2010; Weber and Dan, 2016).

The ascending reticular activating system (Moruzzi and Magoun, 1949) comprises cholinergic, monoaminergic (serotonin, noradrenaline, histamine) and orexinergic nuclei in the brainstem, basal forebrain, and hypothalamus—with wide-ranging projections throughout the entire brain (Luppi, 2010). The hypothalamus also contains key sleep-promoting neuronal populations; in particular, the ventrolateral preoptic area (VLPO) and median preoptic area (MNPO) primarily express the inhibitory neurotransmitters **γ**-aminobutyric acid (GABA) and galanin, and project to all major hypothalamic and brainstem nuclei of the wake-promoting system (Sherin et al., 1996). Homeostatically arranged, the sleep-active neurons of the preoptic hypothalamus are in turn inhibited by the wake-active nuclei they target, especially those of predominantly noradrenergic and serotonergic transmitter phenotype (Gallopin et al., 2000; Chou et al., 2002). This architecture of mutually inhibitory wake-promoting and sleep-promoting circuits constitutes what is known as a “flip-flop switch” (Saper et al., 2001, 2005, 2010): a bistable system characterised by sharp transitions between its two possible states. Damage to the wake-promoting system causes excessive sleep, while insomnia results from damage to the VLPO (Economo, 1930; Lu et al., 2000). In addition to their sleep-promoting effects, VLPO neurons have also been implicated in the mechanisms of action of anaesthetic drugs (Moore et al., 2012; Zhang et al., 2015). Of note, recent evidence also indicates a common role of hypothalamic neuroendocrine cells of the mouse in sleep generation and general anaesthesia induced by several different anaesthetics, with opto- or chemo-genetic activation of these cells promoting both slow-wave sleep and anaesthesia, and the opposite result obtained by inhibiting them (Jiang-Xie et al., 2019).

Current theories propose that at least some anaesthetic drugs may exert their effect by recruiting the brain’s endogenous mechanisms for the production of unconsciousness (Franks, 2008; Alkire et al., 2009; Scharf and Kelz, 2013; Van Swinderen and Kottler, 2014; but see Vanini et al., 2020, for a recent suggestion that this may not be the case, for isoflurane). This may occur through activation of the sleep-promoting pathways, inhibition of the wake-promoting ones, or both [especially since, given their mutually inhibitory nature, activating one will also result in inhibition of the other (Pace-Schott and Hobson, 2002)].

### Neural Inertia and Sleep Inertia

#### Neural Inertia

**FIGURE 2:**
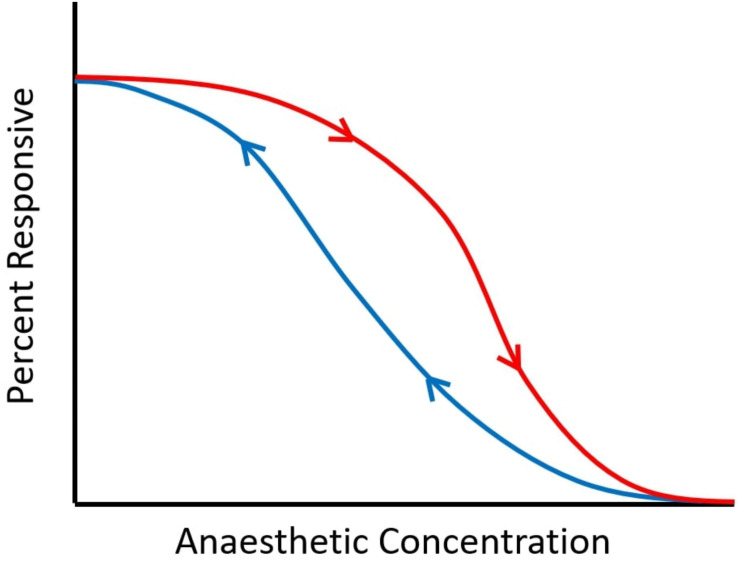
Schematic of neural inertia. As anaesthetic dose is increased, responsiveness is diminished. However, the dose at which a certain proportion of responses is observed is not the same for induction (downward arrow, in red) and emergence (upward arrow, in blue), indicating path-dependence (hysteresis). Between the two curves, subjects may be awake or anaesthetised, depending on whether the drug concentration is being increased or decreased. The wider the gap between the two curves, the greater the hysteresis.

“Neural inertia” refers to the brain’s tendency to resist changes in its arousal state: it is manifested as emergence from anaesthesia (recovery of responsiveness, ROR) occurring at lower drug doses than those required for anaesthetic induction (loss of responsiveness, LOR) (Friedman et al., 2010). Thus, for intermediate dosages between those required for ROR and LOR, a given individual may be anaesthetised or awake, depending on their previous state. This “path dependence” (referred to as hysteresis in physics; Figure 2) is in contrast with pharmacokinetic-pharmacodynamic accounts, which assume that anaesthetic state is fully determined by current effect-site concentration of anaesthetic (McKay et al., 2006).

Rather, evidence of hysteresis between anaesthetic induction and emergence obtained in mice and *Drosophila* led to the proposal that the brain has a tendency to resist transitions in its arousal state, called “neural inertia” (Friedman et al., 2010). Indeed, bistable systems—of which the brain appears to be one, with respect to its sleep-wake states (Saper et al., 2001)—tend to show distinct non-overlapping paths between their states, indicating hysteresis (Chatterjee et al., 2008). Consistent with theoretical work on anaesthesia (Steyn-Ross et al., 2004), this is precisely what Friedman and colleagues observed with regard to anaesthetic induction and emergence in both mammals and invertebrates (Friedman et al., 2010). However, evidence for neural inertia in humans is less clear-cut (Sepúlveda et al., 2019) since it is not possible to measure anaesthetic concentration in the brain in the same way this is commonly done in animal models. Sepulveda and colleagues (Sepúlveda et al., 2018) found that LOR occurred at greater propofol concentrations than ROR, but noted that this result may be alternatively explained by incomplete equilibration between plasma and effect-site concentrations. A different team of researchers (Kuizenga et al., 2018) did not find evidence of neural inertia with propofol, whereas they did observe it with sevoflurane, when combined with the opioid remifentanil. These authors also observed that the choice of marker (behavioural endpoint) with respect to which to compute differences in drug concentration at induction and emergence (e.g., loss and recovery of responsiveness, or EEG features) may also make a difference in investigators’ ability to detect evidence of neural inertia (Kuizenga et al., 2018).

In line with this observation, Warnaby et al. (2017) reported hysteresis for the prevalence of slow-wave EEG activity for both propofol and sevoflurane, with or without addition of opioids; slow-wave persistence was therefore proposed as a marker of neural inertia in humans. While some authors (Colin et al., 2018) criticised this study by arguing that the hysteresis observed by Warnaby and colleagues can be collapsed if a different effect-site equilibration model is assumed, recent modelling work by Proekt and Kelz (2020) demonstrated that—since effect-site concentration is a theoretical construct that cannot be measured directly—it is experimentally impossible to distinguish between an equilibration model that collapses hysteresis and one that does not, even when hysteresis is in part attributable to genuine neuronal dynamics. Therefore, although it is clear that improved methodologies will be required (Proekt and Kelz, 2020), there is reason to believe that humans may also be subject to neural inertia—a postulation consistent with the unequivocal evidence that neural inertia is a widespread phenomenon observed in species as diverse as fruit flies, zebrafish, and rodents (Sepúlveda et al., 2019; Wasilczuk et al., 2020). As Proekt and Kelz observe: *“whereas going from the structured to the unstructured state is trivial, the restoration of structure is not generically expected after a dramatic perturbation*” (Proekt and Kelz, 2020). Thus, emergence may be an active rather than passive phenomenon, the understanding of which will likely need to invoke specific and distinct neurobiological mechanisms beyond a mere reversal of the induction process.

#### Sleep Inertia

Transitions in the brain’s arousal state do not occur only after anaesthesia, but also after sleep. Familiar to many people, this state of transition between sleep and wakefulness, characterised by low levels of arousal and vigilance, sleepiness, confusion, and a temporary reduction in performance, is called sleep inertia (SI) (Tassi and Muzet, 2000; Voss, 2010; Trotti, 2017). Sleep inertia dissipates with time awake, with estimates of its typical duration ranging from 20 to 30 min (Dinges et al., 1987; Tassi et al., 1992) to 1–2 h post-awakening (Jewett et al., 1999). Although sleep inertia occurs even in the absence of sleep debt (Akerstedt and Folkard, 1997), its effects are more profound and long-lasting after a period of sleep deprivation (Ferrara and De Gennaro, 2000). Finally, waking up from slow-wave sleep appears to have the most profound negative impact on subsequent vigilance and performance (Dinges, 1990; Bonnet, 1993; Matchock and Mordkoff, 2014).

From a behavioural perspective, sleep inertia affects performance in the same way as sleepiness (Balkin and Badia, 1988). The human electroencephalographic (EEG) signatures of sleep inertia are also analogous to what is observed at increased levels of sleepiness (Voss, 2010). For approximately 10 min post-awakening, EEG is characterised by elevated low-frequency (1–9 Hz) and reduced beta (18–25 Hz) power (Ogilvie and Simons, 1992; Ferrara et al., 2006; Marzano et al., 2011). Analogous results have been obtained in rodents using intracranial recordings during the first 10 min post-sleep: neuronal activity was low upon awakening, with brief periods of neuronal silence (Vyazovskiy et al., 2014). Crucially, such population OFF periods are typically observed not only during sleep, but also after prolonged wake, as revealed by intracranial recordings in rats (Vyazovskiy et al., 2011). Likewise, recordings in monkeys transitioning from wake to sleep show sleep-like patterns of activity in their visual cortex, even while performing a visual task (Pigarev et al., 1997). Thus, across species sleep inertia appears to be the post-sleep counterpart of pre-sleep sleepiness, with both states characterised by similar behavioural changes and EEG signatures, as well as local sleep-like OFF periods.

#### Neural Inertia as the Effect of Sleep Inertia

Single-gene mutations that increase or decrease neural inertia also affect the sleep-wake cycle, pointing to a connection between anaesthesia, neural inertia and sleep in both invertebrates and mammals (Friedman et al., 2010; Joiner et al., 2013). Here, we propose that neural inertia—the reduction in anaesthetic dose required for emergence compared to induction—may be an effect of the sleep inertia that follows anaesthetic-induced sleep. Specifically, GABA-ergic anaesthetics such as propofol and the inhalational agents sevoflurane, isoflurane, and halothane are believed to induce a state of artificial sleep (Brown et al., 2010; Van Swinderen and Kottler, 2014; but see Vanini et al., 2020). Like natural sleep, this artificial sleep should then be followed by sleep inertia—especially for intravenous drugs such as propofol that induce an artificial sleep characterised by high levels of slow-wave activity (SWA) (Brown et al., 2011; Murphy et al., 2011; Gent and Adamantidis, 2017), since sleep inertia is particularly pronounced upon awakening from slow-wave sleep (Dinges, 1990).

Thus, in the process of emerging from anaesthesia the brain would find itself in the state of sleep inertia, which is behaviourally and neurally equivalent to sleepiness. Since sleepiness is known to increase susceptibility to anaesthesia with propofol, isoflurane, and sevoflurane by lowering the dose that is required for induction, as indicated by rodent studies (Tung et al., 2002; Pal et al., 2011; Scharf and Kelz, 2013), this could explain neural inertia: due to being in a state equivalent to sleepiness, the brain during emergence is more susceptible to anaesthetics than it was at induction, and a smaller dose is sufficient to maintain unconsciousness—producing the hysteresis characteristic of neural inertia.

If this hypothesis is correct, then we predict that neural inertia should be larger when awakening from “recovery sleep” after sleep deprivation, since sleep deprivation increases the sleep inertia that is observed after awakening (Ferrara and De Gennaro, 2000). This is precisely what is observed empirically, with higher neural inertia in previously sleep-deprived animals (Joiner et al., 2013). Moreover, this hypothesis could explain why Friedman and colleagues (Friedman et al., 2010) observed greater neural inertia with halothane than with isoflurane—a result that was recently replicated in mice exposed to equipotent doses of isoflurane, sevoflurane, and halothane, demonstrating that different anaesthetics have different effects on neural inertia, distinct from their potency (Wasilczuk et al., 2020). Specifically, to explain these results we note that unlike isoflurane, halothane does not reduce NREM sleep-debt in rodents (Pick et al., 2011; Scharf and Kelz, 2013). Thus, higher levels of NREM sleep debt would be present upon emergence from halothane than isoflurane, leading to stronger sleep inertia, and hence stronger neural inertia, as observed.

**FIGURE 3:**
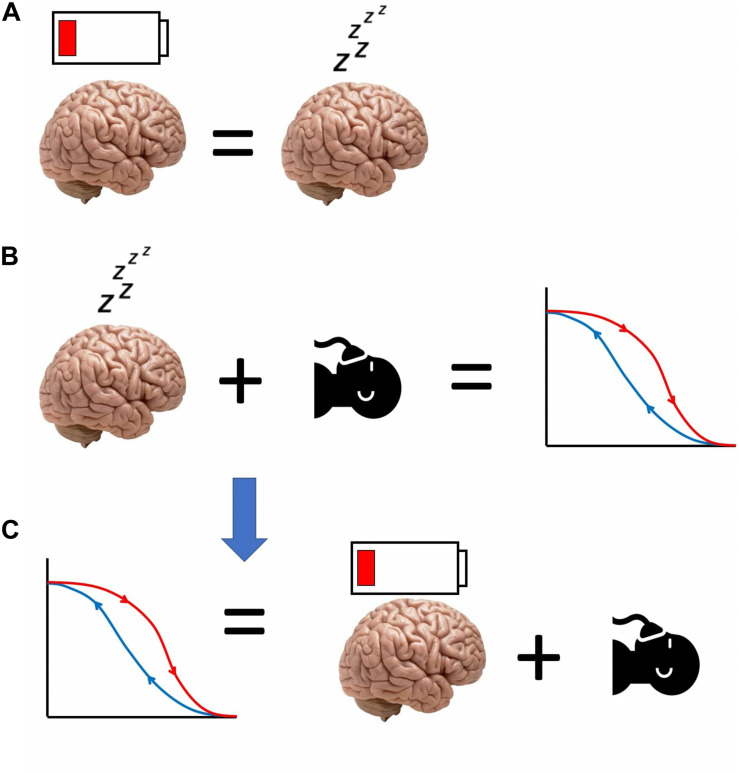
Schematic of our hypothesis equating neural inertia with post-anaesthetic sleep inertia. **(A)** Sleep inertia (represented by the depleted battery icon over the brain) is neurally and behaviourally equivalent to sleepiness. **(B)** Sleepiness reduces the need for anaesthetic, and increases post-anaesthetic neural inertia. Therefore, **(C)** neural inertia may be seen as the manifestation of sleep inertia occurring after anaesthesia, reducing the amount of anaesthetic that is needed for the brain to be unresponsive.

Thus, we have proposed that anaesthesia causes artificial, SWA-rich sleep, which in turn induces sleep inertia. The latter’s effects resemble those of sleepiness, which increases sensitivity to anaesthetics. Therefore, a lower dose of anaesthetic will suffice to keep the brain anaesthetised, resulting in neural inertia at emergence (Figure 3). This hypothesis for the origin of neural inertia could be tested by inducing anaesthesia during the state of sleep inertia, and assessing the prediction that the induction dose will be lower than usual and comparable to the drug level at which emergence typically occurs.

Furthermore, our hypothesis predicts that in the presence of neural inertia, neural activity during emergence should resemble the patterns of sleep-like activity characteristic of sleepiness and sleep inertia—and indeed, there is evidence that slow-wave activity reminiscent of sleep dominates human EEG at the beginning of emergence from anaesthesia, before most patients transition to non-slow-wave activity and subsequent waking (Chander et al., 2014). Additionally, individual measures of susceptibility to sleep inertia could be used to predict individual susceptibility to neural inertia, such as the recently developed Sleep Inertia Questionnaire (Kanady and Harvey, 2015). Indeed, there is already evidence that state-dependent EEG markers at baseline can predict individual susceptibility to anaesthetic induction with propofol (Chennu et al., 2016; Zhang et al., 2020), and future research may seek to determine whether such markers are related to sleep inertia.

We also note that our hypothesis would likely not apply to the dissociative anaesthesia induced by ketamine, whose molecular mechanisms of action and neurophysiological effects at the micro- and macroscale are very different from other known anaesthetics, and do not appear to resemble sleep (Hemmings et al., 2019). Although we are not aware of tests of neural inertia with ketamine, our hypothesis leads us to predict that little should be observed, since sleep does not seem to be involved in the context of dissociative anaesthesia. Testing this prediction in humans is not straightforward, for the same reason that complicates existing attempts to identify neural inertia in humans (Sepúlveda et al., 2019): namely Proekt and Kelz (2020) demonstrated that since effect-site concentration cannot be measured directly, effect-site models could be constructed to collapse hysteresis even when it would actually be attributable to genuine neuronal dynamics. However, the hypothesis is not specific to humans and could be tested in other species for which neural inertia has already been demonstrated with other anaesthetics (Friedman et al., 2010; Joiner et al., 2013; McKinstry-Wu et al., 2019; Wasilczuk et al., 2020), with the prediction being that little hysteresis should be observed. Additionally, we reported above that if neural inertia is due to the increased susceptibility to anaesthetics that occurs during post-anaesthetic sleep inertia, then our hypothesis predicts that higher susceptibility to anaesthesia should be observed during sleep inertia (e.g., as induced by awakening from slow-wave sleep). We expect that ketamine would constitute an exception to this general prediction—which should be testable in humans.

Recently, a modelling study observed that neural inertia is compatible with an account of the brain as a bistable system, stochastically switching between two states (Proekt and Hudson, 2018). If the states are seen as wells in an energy landscape, the system can be conceptualised as transitioning between them whenever noise-driven (stochastic) fluctuations are large enough to overcome the energy differential between the wells. Under conditions of low noise, the system is therefore more likely to remain trapped in whatever state it is currently occupying, and therefore inertia (resistance to state transitions) will be observed (Proekt and Hudson, 2018). It is important to note that our hypothesis of neural inertia as the effects of sleep inertia arising from anaesthetic-induced “artificial sleep” is not incompatible with this account of neural inertia: the two operate at different levels of explanation (Marr, 2010). In fact, if our hypothesis is correct, then it suggests that the account of Proekt and Hudson (2018) could also be invoked to understand sleep inertia.

If corroborated, the hypothesis presented here could have direct relevance for clinical practice: anaesthetists could use tools such as the recently developed Sleep Inertia Questionnaire (Kanady and Harvey, 2015) to evaluate each patient’s individual susceptibility to sleep inertia, which we expect should predict (together with their current amount of sleep debt) their individual likelihood of experiencing neural inertia.

#### Neuroimaging Evidence: Diminished Anticorrelations in the Inert Human Brain

**FIGURE 4:**
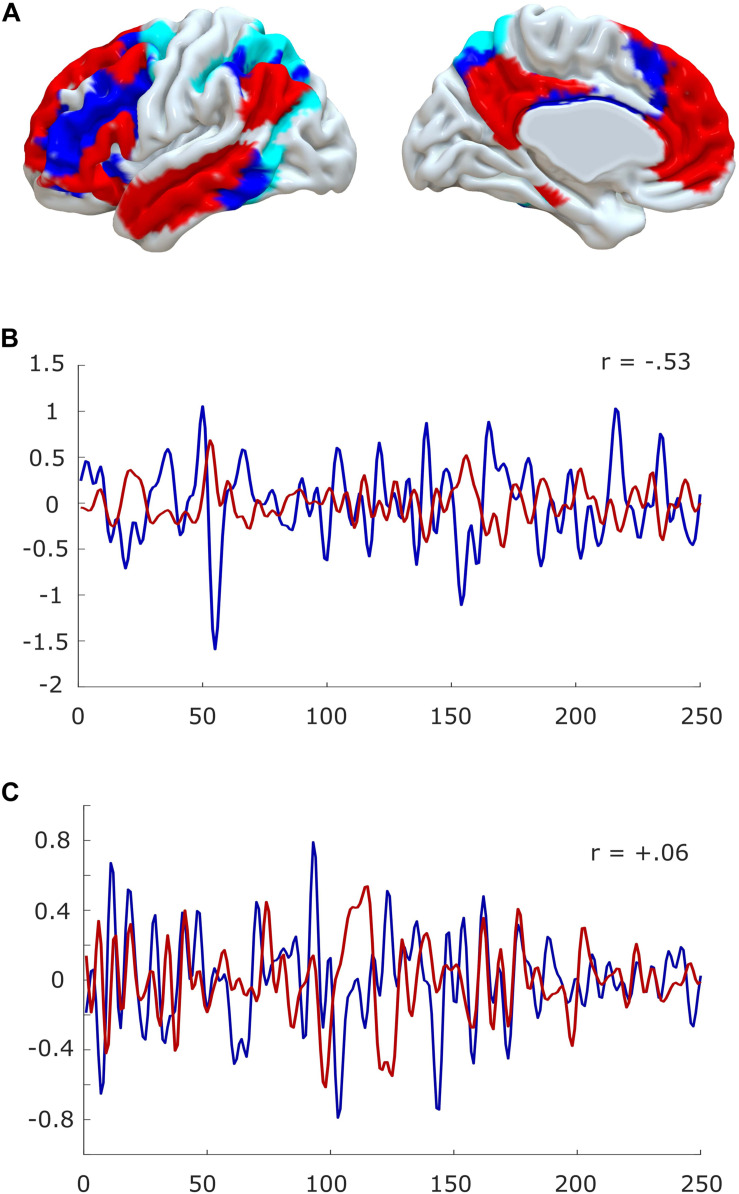
Anticorrelations in the human brain. **(A)** Surface projection of the default mode network (red) and fronto-parietal (blue)/dorsal attention networks (cyan) onto medial and lateral surfaces of a standard brain (left hemisphere). **(B)** The timecourses of default mode and fronto-parietal networks are anti-correlated during quiet wakefulness. **(C)** Anticorrelations are reduced or even abolished in the anaesthetised brain. Data from one representative subject, before and during propofol anaesthesia; for experimental details, see Stamatakis et al. (2010) and Varley et al. (2020).

At the macroscale, there is additional recent evidence to suggest that anaesthesia resembles the state of sleep inertia. Under conditions of normal restfulness, it is well known from functional MRI that the human brain self-organises into distinct sets of brain regions, known as resting-state networks (Yeo et al., 2011; Smith et al., 2012). In particular, a “default mode” network (DMN) of medial frontal and parietal regions, and a set of “task-positive” networks such as the “executive control” network of lateral fronto-parietal regions (FPN) and the “dorsal attention network” (DAN) tend to exhibit anticorrelated patterns of activation (Raichle et al., 2001; Fox et al., 2005) (but note that the DMN can also be recruited by tasks, especially pertaining to self-referential cognition, “mental time travel,” or automated processing (Vatansever et al., 2015a,b, 2017; Buckner and DiNicola, 2019) (Figure 4).

Intriguingly, recent EEG-fMRI evidence indicates that loss of DMN-FPN/DAN anticorrelations is a neural correlate of sleep inertia itself in humans (Vallat et al., 2018; but see Chen et al., 2020). Indeed, earlier work had also demonstrated, by employing positron emission tomography (PET) that for a short period of time after awakening (5–20 min, compatible with the duration of sleep inertia; Trotti, 2017), there is a gradual increase of cerebral blood flow in heteromodal areas, especially lateral prefrontal cortex (lPFC), a core component of the executive control network (Balkin et al., 2002). Additionally, as previously mentioned, both awakening from deep sleep and previous sleep deprivation intensify subsequent sleep inertia upon awakening. And indeed, a loss of DMN-FPN/DAN anticorrelations is also observed during sleep in humans (Sämann et al., 2011), as well as in the awake but sleep-deprived human brain (De Havas et al., 2012). Thus, sleep inertia and conditions that favour it, share a common neural substrate in the reduction of DMN-FPN/DAN anticorrelations. Conversely, caffeine consumption, perhaps the most widely adopted countermeasure to sleep inertia (Van Dongen et al., 2001) is known to have the opposite effect: it increases the anticorrelations between DMN and FPN/DAN in the human brain (Wong et al., 2012).

This suggests that sleep inertia, at least in the human brain, may correspond to a carry-over of diminished DMN-FPN/DAN anticorrelations. Remarkably, perturbed DMN-FPN/DAN interactions are also one of the most robustly observed neural markers of human loss of consciousness induced by a variety of anaesthetics (Boveroux et al., 2010; Guldenmund et al., 2013; Golkowski et al., 2019; Luppi et al., 2019, 2020; Huang et al., 2020) (Figure 4), and the anticorrelations are even diminished one hour after emergence from sevoflurane anaesthesia (Nir et al., 2020). Thus, we propose that neural inertia may be the effect of anaesthetic-induced sleep inertia, which corresponds to a carry-over of diminished anticorrelations between DMN and FPN/DAN. In other words, we propose that the inert brain is a brain that has lost its characteristic anticorrelations. This specific hypothesis could be empirically tested, since it predicts that humans experiencing higher neural inertia after anaesthesia should exhibit more prominent loss of anticorrelations.

### Inertia in the Aging Brain

Intriguingly, the hypothesis presented here may also explain why older adults are more susceptible to neural inertia (Warnaby et al., 2017). Namely, according to the present view, this is because they are more susceptible to sleep inertia. Reduced and fragmented sleep is common among the elderly, and especially patients with Alzheimer’s disease (Bonanni et al., 2005; Guarnieri et al., 2012). Since fragmented sleep tends to increase subsequent slow-wave activity (Bonnet, 1987), awakening from which causes higher levels of sleep inertia (Dinges, 1990), as does sleep deprivation, the elderly should show higher levels of sleep inertia. This is indeed the case (Silva and Duffy, 2008).

Additionally, if the hypothesis proposed here about the link between sleep inertia and neural inertia is correct, these populations should also suffer from higher levels of neural inertia. Again, this is precisely what is observed: rat studies indicate that ageing increases sensitivity to anaesthetics, and prolongs their effect (Chemali et al., 2015); likewise, older humans are also more susceptible to anaesthesia (Kanonidou and Karystianou, 2007). Furthermore, recent evidence indicates that age influences the newly discovered EEG marker of neural inertia in humans, slow wave activity saturation (SWAS): SWAS is more likely to cease abruptly rather than gradually in older patients, predicting their likelihood of post-operative delirium (Warnaby et al., 2017).

Neuroimaging evidence in older adults further supports the link between sleep and neural inertia and loss of anticorrelations between DMN and FPN/DAN: it is well established that aging corresponds to a reduction of anticorrelations between these networks (Keller et al., 2015; Siman-Tov et al., 2017), even in the absence of concomitant psychiatric conditions (Kobuti Ferreira et al., 2015) and more so in those with mild cognitive impairment (Esposito et al., 2018). Thus, older brains are intrinsically more prone to loss of anticorrelations, and suffer from higher sleep inertia and higher neural inertia.

### Molecular Mechanisms of Sleep and Neural Inertia

#### Orexin/Hypocretin

One candidate system for the control of sleep inertia—and hence, we have argued, neural inertia—is the orexinergic system. Located exclusively in the lateral hypothalamus (De Lecea et al., 1998; Sakurai et al., 1998), orexin/hypocretin neurons are wake-active (Lee et al., 2005; de Lecea and Huerta, 2014), and innervate the wake-promoting monoaminergic and cholinergic nuclei (Carter et al., 2012). And indeed, using channelrhodopsin-2 to selectively stimulate orexin neurons promotes awakening from sleep in mice (Adamantidis et al., 2007), and increased wakefulness is reported in rodents after orexin-A administration, either intracerebroventricular or directly into monoaminergic and cholinergic wake-promoting nuclei (Hagan et al., 1999; Sakurai and Mieda, 2011).

Conversely, optogenetic suppression of orexin neurons with archaerhodopsin has sleep-promoting effects in mice (Tsunematsu et al., 2011, 2013); and in humans, orexin blockers are now available as medication against insomnia (Bennett et al., 2014). These effects were confirmed using Designer Receptors Exclusively Activated by Designer Drugs (DREADDs) to chemogenetically activate or silence orexin neurons, resulting in increased wakefulness or sleep in rodents, respectively (Sasaki et al., 2011). Loss of orexin neurons causes the sleep disorder narcolepsy in dogs (Lin et al., 1999) and humans (Nishino et al., 2000; Thannickal et al., 2000), and the same is obtained by selective orexin knock-out in mice (Chemelli et al., 1999; Mochizuki et al., 2004), as well as pharmacological lesions in rats (Gerashchenko et al., 2001). Crucially, narcolepsy is characterised by an unstable and fragmented sleep-wake cycle, and difficulty in becoming awake (i.e., high sleep inertia) (Scammell, 2003). Indeed, sleep inertia is often present in narcoleptic children (Wise, 1998).

**FIGURE 5:**
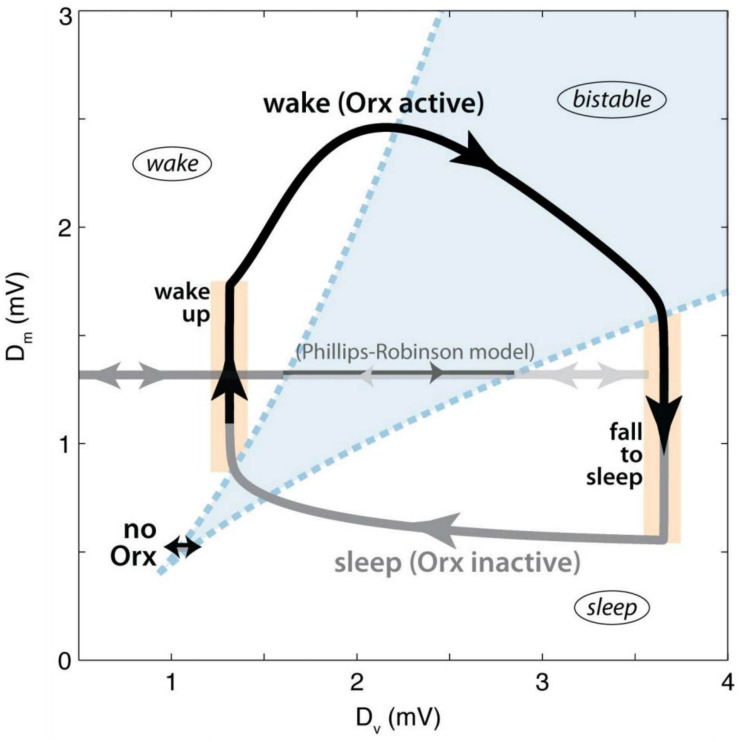
Dynamics of orexin stabilisation of state transitions according to the model of Fulcher et al. (2014). The axes represent the net drives to the wake-promoting (D_*M*_) and sleep-promoting (D_*V*_) circuits. Regions are labelled, with the bistable region shown in blue. The black arrow represents the waking period, while the grey arrow represents sleep. The trajectory marked “no Orx” represents the dynamics of the model in the absence of orexin input. Figure adapted from Figure 3 of Fulcher et al. (2014), published under CC-BY licence.

Thus, there is ample evidence, in both humans and other animals, that orexin and orexinergic neurons play a crucial role in sleep-wake regulation (Mieda, 2017). A recent computational study indicates that the specific role of orexin may be to stabilise the transitions between sleep and wake (Fulcher et al., 2014). According to the model, a bistable region of state-space exists when the inputs to the sleep-promoting and wake-promoting systems are balanced, and state transitions are easy (Figure 5). By increasing the activity of wake-promoting monoaminergic nuclei upon awakening, orexin pushes the system out of the bistable region, stabilising it. Indeed, simulating orexin loss in the model lowered transition thresholds, resulting in frequent wake-sleep transitions and sleep fragmentation, analogous to what is observed in orexin-deficient narcoleptic patients.

Intriguingly, recent neuroimaging work using a variant of functional MRI called MR encephalography, which has high temporal resolution (100 ms), determined that human narcoleptic patients have aberrant interactions between DMN and FPN/DAN, characterised by delayed and monotonic interactions, which the authors interpreted as a compromised ability of task-positive networks to suppress the DMN (Järvelä et al., 2020); Once again, this observation is in line with our proposed macroscale identification of sleep inertia with abnormal anticorrelations between large-scale networks of the brain.

Thus, evidence suggests that low orexin levels lead to high levels of sleep inertia, and its associated neural signatures. According to the hypothesis developed here, such high sleep inertia should be accompanied by high levels of neural inertia. This is indeed the case: case reports suggest high neural inertia in at least some narcoleptic human patients (Mesa et al., 2000; Burrow et al., 2005), confirmed by the increased neural inertia observed in rodents with narcolepsy arising from genetic ablation of orexin neurons (Hara et al., 2001; Kelz et al., 2008). Moreover, orexin is known to be involved in anaesthetic action: the activity of orexin neurons is reduced by propofol, sevoflurane and isoflurane, as indicated by a reduced number of c-Fos-immuno-reactive orexinergic neurons in rodents (Kelz et al., 2008; Zhang et al., 2012; Scharf and Kelz, 2013). Moreover, rodent studies show that reduced activation of orexin neurons during anaesthesia is exacerbated when the anaesthesia is administered under conditions of sleep deprivation (Ran et al., 2015). Conversely, intracerebroventricular administration of orexin-A (though not orexin-B) causes emergence from propofol, isoflurane and sevoflurane anaesthesia in rats (Dong et al., 2009; Shirasaka et al., 2011; Zhang et al., 2012, 2016), and similar results have also been obtained in mice, whereby activation of orexin neurons with DREADDs facilitated emergence from isoflurane anaesthesia (Zhou et al., 2018). Thus, orexin appears to play a major role in anaesthesia and the sleep-wake cycle, with its absence increasing both sleep inertia and neural inertia.

#### Noradrenaline

The action of orexin neurons is believed to occur mainly through excitation of monoaminergic wake-promoting nuclei, which they innervate (Sakurai and Mieda, 2011). In particular, orexinergic neurons may exert their effects on sleep-wake transitions through the noradrenergic *locus coeruleus* (LC) (Carter et al., 2012). Orexin neurons send strong excitatory projections to the LC, and the wake-inducing effect of orexin infusion involves activation of the LC (Hagan et al., 1999).

Indeed, the fragmented sleep-wake cycle of narcolepsy was reconsolidated by restoring orexin receptors in the LC of mice, and equivalent results were achieved by chemogenetically activating these neurons with DREADDs (Hasegawa et al., 2014). Furthermore, optogenetic inactivation of LC prevents the arousal-promoting effect of optogenetically activating orexin neurons; conversely, the latter is potentiated by concomitant stimulation of LC neurons (Carter et al., 2012). Thus, there is strong evidence that noradrenergic system activity is one of the primary routes through which orexin neurons perform their regulatory role (De Lecea, 2015).

Specifically supporting a role for noradrenaline in neural inertia, previous work (Friedman et al., 2010) established that genetic deletion of dopamine-ß-hydroxylase (DBH) in mice to remove noradrenergic signalling resulted in hypersensitivity to isoflurane anaesthesia, as well as increased neural inertia. This could be reversed by pharmacologic CNS-specific rescue of adrenergic signalling, achieved by providing the amino acid L-DOPS so that it would be converted into noradrenaline by L-amino acid decarboxylase (Friedman et al., 2010). In humans, Kuizenga et al. (2018) reported evidence of neural inertia when sevoflurane was supplemented with remifentanil, which is believed to influence sleep-wake regulation through adrenergic neurotransmission (McCormick and Bal, 1997; Samuels and Szabadi, 2008).

Indeed, implication of orexin and noradrenaline in neural inertia has been considered before (Sepúlveda et al., 2019; Wasilczuk et al., 2020). Wasilczuk et al. (2020) observed that halothane does not suppress hypothalamic orexinergic neurons and LC noradrenergic neurons (Gompf et al., 2009), whereas isoflurane does suppress them (Kelz et al., 2008). Thus, these authors proposed that this difference may underlie the increased neural inertia induced by halothane compared with isoflurane (Friedman et al., 2010; Wasilczuk et al., 2020) due to non-abolished orexinergic activity. As mentioned above, our own explanation of the same phenomenon is in terms of halothane failing to reduce sleep debt, unlike isoflurane (Pick et al., 2011), thereby producing more sleep inertia (and hence neural inertia, according to our account). These two explanations are not in contrast: indeed, they suggest that a fruitful avenue for future research may be to seek a connection between persistent orexinergic activity and halothane’s failure to discharge sleep debt.

On the other hand, studies providing a direct link between noradrenaline and sleep inertia are presently lacking; nevertheless, several indirect lines of evidence suggest that low levels of noradrenaline may be related to sleep inertia. Behaviourally, noradrenaline is implicated in cognitive functions such as sustained attention and working memory (Chamberlain and Robbins, 2013; Spencer et al., 2015), which are especially vulnerable to sleep deprivation (Goel et al., 2009; Killgore, 2010)—of which sleep inertia is a post-awakening counterpart, we have argued here. Noradrenaline is also increased following consumption of coffee (Papadelis et al., 2003), and caffeine consumption can reverse many of the cognitive adverse effects of clonidine (Smith et al., 2003), which mimics the state of reduced arousal observed as a result of sleep deprivation by reducing turnover of central noradrenaline, by binding to autoreceptors (Nutt and Glue, 1988).

Recently, Bellesi et al. (2016) used *in vivo* microdialysis to demonstrate decreasing levels of prefrontal noradrenaline in rodents undergoing sleep deprivation, correlating with an increase in low EEG frequencies tracking the need to sleep. Thus, low levels of prefrontal noradrenaline could contribute to explain the cognitive deficits observed during sleepiness induced by prolonged wakefulness. Crucially, noradrenaline restoration to baseline levels post-awakening was slower in prefrontal cortex than in other areas, such as M1—and in humans, prefrontal regions are those that were found to have reduced cerebral blood flow upon awakening in the PET study of Balkin et al. (2002). Thus, evidence suggests that decreased prefrontal noradrenaline could also explain the confusion and cognitive deficits observed during sleep inertia—especially since this state is very similar to sleepiness, as we have shown. This evidence also suggests that, if our hypothesis is correct, then we should expect noradrenaline to modulate the prevalence of anticorrelations between DMN and FPN/DAN in the human brain, since anticorrelations are also enhanced by caffeine and decreased by sleepiness (De Havas et al., 2012), sleep (Sämann et al., 2011), sleep inertia (Vallat et al., 2018), and anaesthesia (Boveroux et al., 2010; Golkowski et al., 2019; Luppi et al., 2019; Huang et al., 2020). Interestingly, recent studies indicate that caffeine infusion can accelerate emergence from isoflurane anaesthesia in both rodents and humans (Fong et al., 2018; Fox et al., 2020), and future research may seek to determine whether this effect corresponds to faster recovery of anticorrelations in the brain after anaesthesia (Nir et al., 2020) and whether it is specifically attributable to caffeine’s action on noradrenergic neuromodulation (Papadelis et al., 2003; Smith et al., 2003).

Indeed, as major wake- and alertness-promoting neurotransmitter, noradrenaline is modulated by both sleep and anaesthesia—just as we should expect if noradrenaline were involved in both sleep and neural inertia, as we propose here. Noradrenaline levels are highest during wake and drop during sleep (Léna et al., 2005) and stimulation of the noradrenergic LC of mice induces waking (Carter et al., 2010, 2013; Berridge et al., 2012)**;** activity of the LC is inhibited by GABA during sleep (Gervasoni et al., 1998), as well as during propofol and isoflurane anaesthesia in mice (Zecharia et al., 2009). Administration of noradrenaline by microinjection into the central medial nucleus of the thalamus accelerates emergence from propofol anaesthesia in rodents, and reverses the local physiological effects of propofol (Fu et al., 2016). Likewise, pharmacogenetic activation of noradrenergic neurons in the LC with virally delivered DREADDs promotes EEG markers of neural arousal and accelerates emergence from isoflurane anaesthesia in rats, an effect that can be prevented by application of noradrenergic antagonists (Vazey and Aston-Jones, 2014). The anaesthetic dexmedetomidine also operates on noradrenergic transmission: as an adrenergic α-2 receptor agonist, it decreases the firing of LC neurons (Nelson et al., 2003), and indeed α-2A receptor activation inhibits noradrenergic LC neurons (Lakhlani et al., 1997).

Although it was originally thought that dexmedetomidine would induce sedation by inhibiting the LC (Sanders and Maze, 2012) thereby removing the noradrenergic inhibition on the sleep-promoting VLPO neurons (Nelson et al., 2003), recent evidence suggests a more intricate picture: acute inhibition of LC neurons does not induce strong sleep in mice (Carter et al., 2010), and LC inhibition is not required for low doses of dexmedetomidine to produce their sedative effects, since knockdown of LC α2A adrenergic receptors in mice does not prevent sedation, even though loss of the righting reflex is still observed at high doses (Zhang et al., 2015). Intriguingly, the same hypothalamic neurons in the mouse are involved in inducing recovery sleep and dexmedetomidine-induced sedation, by locally exciting neurons in the preoptic area (Zhang et al., 2015).

Other studies also indicate a more complicated picture: microdialysis of noradrenaline into rat prefrontal or parietal cortex under constant levels of sevoflurane anaesthesia failed to produce wake-like behaviour—although it did produce wake-like EEG (Pal et al., 2018). Similar failure to awaken rats from continuous sevoflurane anaesthesia was also reported after pharmacological blockade of noradrenaline reuptake (Kenny et al., 2015). Since cholinergic stimulation of prefrontal cortex did induce wake-like behaviour in the rats studies by Pal et al. (2018), this evidence suggests that a full picture will likely need to also take additional neuromodulatory systems into account. Dopamine in particular has been implicated, largely in rodent studies. Lesions to the wake-active dopaminergic ventral tegmental area in the brainstem shorten the induction time of anaesthesia, and lengthen the time taken for recovery—whereas both electrical and optogenetic stimulation of the VTA can reverse the anaesthetic effects of propofol in rats and mice (Solt et al., 2014; Taylor et al., 2016). These contributions of dopaminergic signalling have recently also been extended to a dopaminergic population in the ventral periaqueductal grey (Li et al., 2018; Liu et al., 2020). Given the shared pathways of dopaminergic and noradrenergic transmitter production, it seems plausible that these transmitters and their nuclei in the brainstem may act in-concert to produce wakefulness, and to counter the effects of sleep inertia and neural inertia, as evidenced by their influences on recovery and induction times. Likewise, the recent discovery that hypothalamic neuroendocrine cells are involved in both slow-wave sleep and general anaesthesia induced by multiple classes of anaesthetic drugs (Jiang-Xie et al., 2019) suggests that a fuller understanding of the link between sleep and neural inertia may benefit from taking into account neuroendocrine involvement.

## Discussion

Overall, there is converging human and animal evidence that neural inertia strongly resembles sleep inertia, in terms of both behavioural manifestations and microscale and macroscale neural markers. Both phenomena are influenced by orexin neurons, which seem to perform a state-stabilising function via noradrenergic transmission. Loss of orexin neurons in narcolepsy, results in fragmented sleep-wake cycles and increases in both sleep inertia and neural inertia. Therefore, we have argued here that neural inertia may in fact be a manifestation of sleep inertia, as it occurs after the artificial slow-wave sleep induced by anaesthetics. Of note, this hypothesis can account for phenomena as diverse as the higher inertia-inducing properties of halothane vs. isoflurane (Friedman et al., 2010; Wasilczuk et al., 2020), and the increased susceptibility to neural inertia in the elderly and in narcoleptic patients.

If our hypothesis is correct, then it could have implications for clinical practice: by assessing each patient’s individual susceptibility to sleep inertia and current sleep debt, anaesthetists may be able to estimate individual likelihood of their patient experiencing neural inertia. In turn, this may better equip them to counteract adverse effects such as post-anaesthetic delirium (Warnaby et al., 2017; Sepúlveda et al., 2019).

Multiple sources of evidence—behavioural and neurophysiological, in animals and humans—suggest that orexin may play a stabilising effect between states of sleep and wakefulness, possibly (though likely not exclusively) through its effects on locus coeruleus noradrenergic neurons. Together, these neuromodulatory systems may be key in determining sleepiness, sleep inertia and what we have argued is its post-anaesthetic counterpart: neural inertia. Nevertheless, direct evidence explicitly linking all pieces of this puzzle together is still lacking, and even evidence of a link between noradrenergic modulation and sleep inertia is at present only indirect. Further studies explicitly investigating involvement of noradrenaline and other neuromodulators in relation to sleep inertia remain necessary, as a test of the hypothesis presented here.

Of course, the brain is a remarkably complex system. There are other components of the sleep- and wake-promoting circuits beyond orexin and noradrenaline, and they are likely to play some direct or indirect role in the phenomena of sleep inertia and neural inertia, and the stabilisation of arousal states more broadly. All these circuits are intricately interconnected, and changes in one are likely to have multiple repercussions. Indeed, investigating such interactions will be required to further elucidate the hypothesis proposed here. Nevertheless, here we have provided a number of predictions that are testable with current scientific techniques, and we hope that these predictions will stimulate fruitful avenues for further research—whether or not they ultimately support our hypothesis.

## Data Availability Statement

The original contributions presented in the study are included in the article, further inquiries can be directed to the corresponding author.

## Author Contributions

AL: conceptualisation and writing—original draft. LS and ES: writing—editing. ES: supervision. ES and DM: project administration and funding acquisition. All authors contributed to the article and approved the submitted version.

## Conflict of Interest

The authors declare that the research was conducted in the absence of any commercial or financial relationships that could be construed as a potential conflict of interest.
